# A novel missense variant of *FBN1* gene in a Sardinian family with Marfan syndrome: a case report

**DOI:** 10.3389/fped.2025.1549504

**Published:** 2025-03-07

**Authors:** Marina Marsan, Mattia Brutti, F. Meloni, M. Marica, C. Soddu, F. Lai, D. Martorana, S. Savasta

**Affiliations:** ^1^ Pediatric Clinic and Rare Diseases, “Microcitemico Hospital”, Cagliari, Italy; ^2^ Unit of Oncology and Molecular Pathology, Department of Biomedical Sciences, University of Cagliari, Cagliari, Italy; ^3^ Medical Genetics Unit, University Hospital Parma, Parma, Italy; ^4^ Pediatric Clinic and Rare Diseases, “Microcitemico Hospital”, Department of Medical Science and Public Health, University of Cagliari, Cagliari, Italy

**Keywords:** case report, *FBN1*, Marfan syndrome, novel variant, pediatrics

## Abstract

**Background:**

Marfan Syndrome (MS) is a connective tissue disorder, an autosomal dominant condition mostly caused by variants in the *FBN1* gene, which encodes for fibrillin-1 protein. Anomalies in the gene lead to a wide variety of clinical manifestations, including disorders of the cardiac, ocular and musculoskeletal system. We present a case of a child belonging to a Sardinian family of four generations, with a novel variant found in the *FBN1* gene.

**Objective:**

To include this novel missense *FBN1* variant into genetic counselling for Marfan Syndrome and to discuss its genotypic-phenotypic correlation.

**Methods:**

Firstly, the proband was diagnosed with Marfan Syndrome using 2020 Revised Ghent Criteria, and she then underwent genetic testing using Next Generation sequencing.

**Results:**

The NGS revealed a novel heterozygous missense variant (c.2348A>G) in the *FBN1* gene, in exon 20. This genetic variant caused a missense substitution of a serine residue with an arginine residue in the position 783 of Fibrillin-1 protein. The variant was then evaluated in the other family members, and was eventually only found in symptomatic individuals, regardless of the severity of their phenotype, demonstrating the segregation with MS; furthermore, it showed complete penetrance with the disease.

**Conclusions:**

Our results suggest that this variant is responsible for MS and it therefore should be included in genetic diagnoses and counselling discussion.

## 1 Introduction

Marfan syndrome (MS) is one of the most common inherited disorders of connective tissue. It has an estimated incidence of 1 in 3,000–5,000 individuals and a prevalence of 1 in 10,000–20,000 individuals without geographic, ethnic or gender predilection (1). In 1896 Antoine-Bernard-Jean Marfan described the first case. The involvement of the aorta in the syndrome was recognized in 1943 (2), and Hollister et al. demonstrated the fibrillin-1-related pathogenesis in 1990 (3).

Inheritance is predominantly autosomal dominant with complete penetrance and considerable clinical variability. A “*de novo*” variant can be found in 25% of cases, mostly (90%) associated with mutation of *FBN1* gene on chromosome 15q21.1, which encodes protein fibrillin-1 (4, 5). This protein is one of the main components of elastic matrix microfibrils, and it is especially present at the cardiovascular and musculoskeletal level (6). Fewer cases are due to mutations of other genes, such as *TGFBR1* or *TGFBR2*.

Point mutations are the most common: 60% missense and 10% nonsense (7, 8). Less frequent mutations consist of small insertions, deletions or duplications (10%–15%) or various classes of splicing errors (10%–15%). Finally, few larger rearrangements have been reported (9), while whole gene deletions are rare (10).

Although specific genotypes do not seem to be related to specific phenotypes (7, 9, 11), variants in exons 24–32 of the *FBN1* gene seem to be characterized by the most severe prognosis: the clinical onset is immediately after birth and death usually occurs within the second year of life (7, 12). Moreover, *TGFBR2* mutations seem to be associated with worse cardiovascular involvement and minor ocular involvement (13). Finally, as well as Marfan Syndrome, other fibrillinopathies might carry variants in the *FBN1* gene, and a differential diagnosis should frequently be considered (7, 8).

Our case report focuses on a novel missense variant located in exon 20 of *FBN1* gene which was found in a family of four generations; inheritance was autosomal dominant and had complete penetrance, whereas phenotype varied significantly among family members.

## 2 Case description

**Figure 1:**
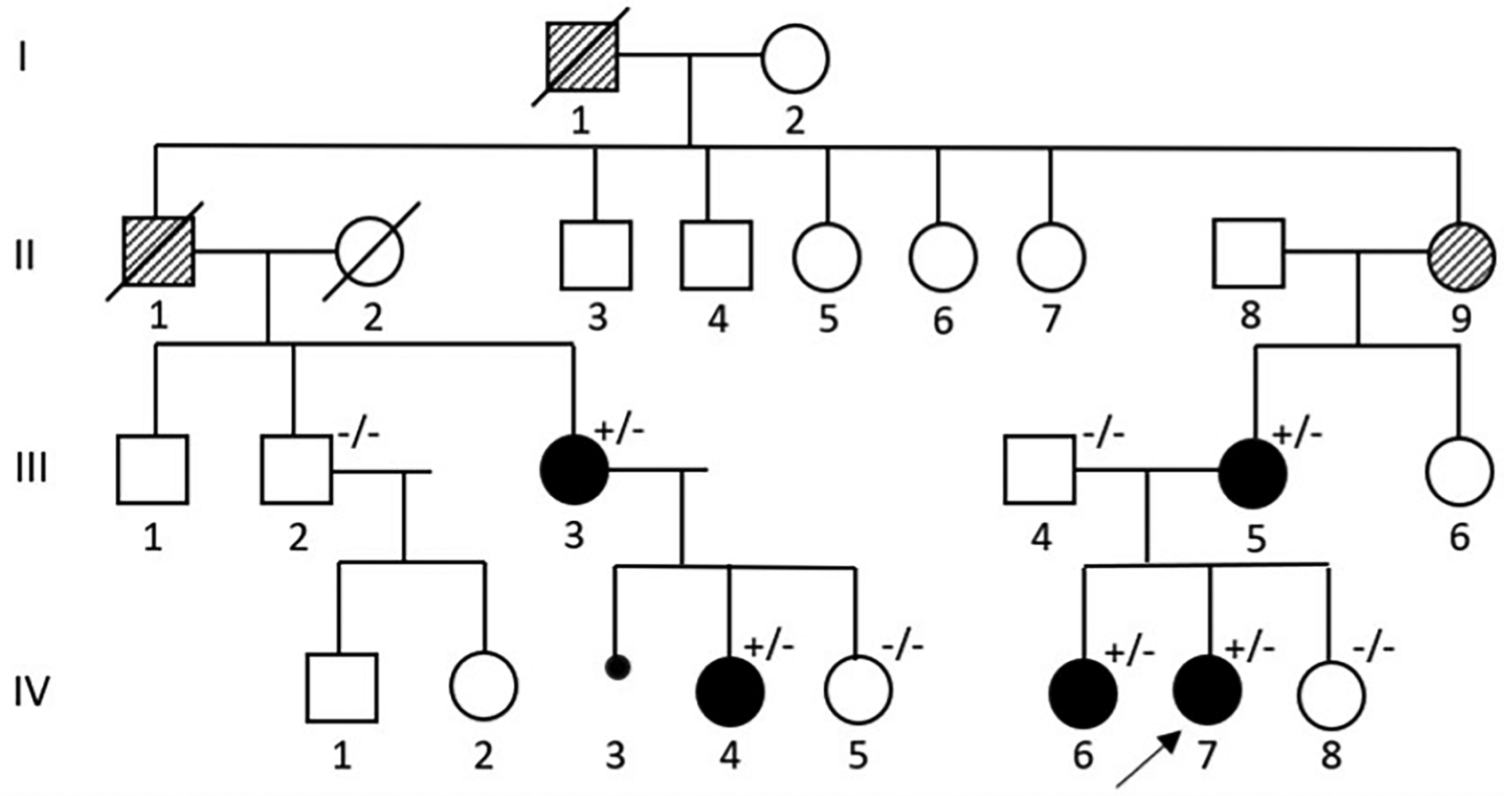
Pedigree of a Sardinian family with Marfan syndrome. The proband is indicated with a black arrow. (+/−): variant carrier and symptomatic. (−/−): not variant carrier and asymptomatic.

We present the case of a family (see Figure 1), located in Sardinia, whose members showed Marfan-related clinical manifestations, which varied both in phenotype as well in different levels of organ involvement.

IV-7 was the first patient to come to our attention. At 8 years of age, she presented with mild scoliosis, bilateral “*pes cavus*”, aortic root diameter at the upper limits and bicuspid aortic valve with mild insufficiency. At clinical examination she had an arm span-height ratio of 0.98, joint laxity with a Beighton score >5, no major facial deformities with the exception of anteverted auricles, and a 2/6 mesosystolic heart murmur. In the following years she had a worsening of the lumbar scoliosis, that required a corrective orthopedic surgery, an increase in the arm span-height ratio, positive thumb and wrist signs, elastic skin, hallux valgus, while the aortic ectasia paralleled the general growth of the patient (*z*-score being 2.99 in 2019, 3.34 in 2020, 3, 7 in 2022); she was therefore started on beta-blockers and angiotensin receptor blockers (ARBs) to minimize aortic dilatation.

**Figure 2:**
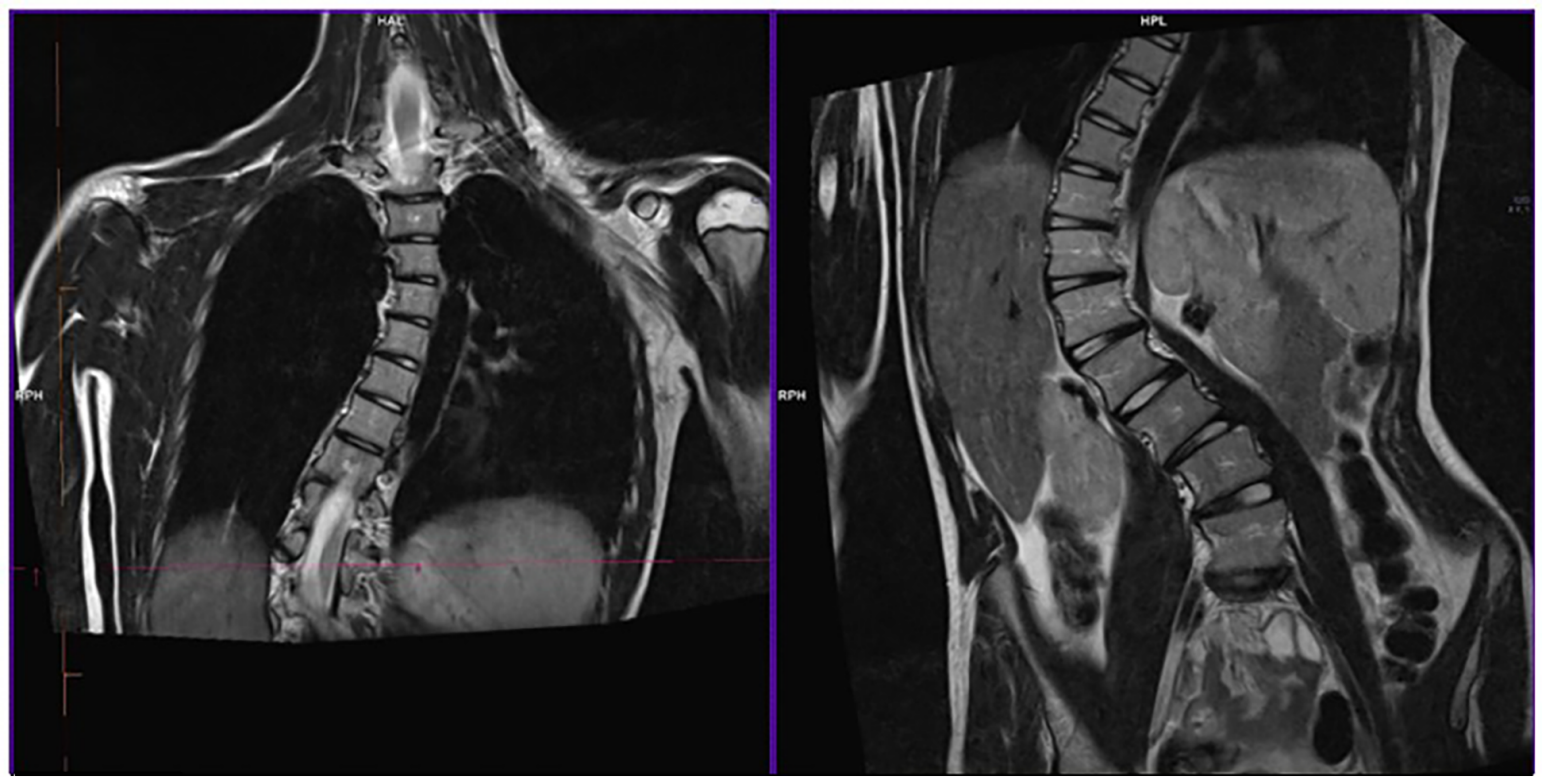
RMI of IV-6's dorsal spine tract before surgery. The image shows dorso-lumbar scoliosis with a 65° Cobb angle.

Her older sister, IV-6, presented with a progressing dorso-lumbar scoliosis with a 65° Cobb angle (see Figure 2), which was diagnosed when she was 10 years old. She then developed an aortic root ectasia at the upper limits, which is being currently treated with anti-hypertensive medication, mitral valve with irregular, redundant thickened and prolapsed flaps, with slight insufficiency and tricuspid valve with mild-moderate secondary insufficiency. Physical examination pointed out an arm span-height ratio of 1.05, arachnodactyly, reduced elbow extension, positive wrist and thumb signs, flat foot, cutaneous striae at the hips, geographic tongue, systolic murmur, reduced vesicular murmur on the right side of the chest because of the scoliosis. Meeting the Revised Ghent criteria (8 total points) she was diagnosed with Marfan' syndrome.

Their mother, III-5, has a mild aortic valve insufficiency and a history of scoliosis treated with back-brace at 15 years old. III-5's cousin, III-3, has a marfanoid habitus, while her daughter, IV-4, of 11 years of age, showed mild ligamentous laxity, arm span-height ratio of 1,03, cutaneous striae on the hips, positive thumb sign, kyphotic appearance, mild scoliosis, bilateral flat foot.

IV-7 and IV-6's clinical signs and symptoms prompted the execution of a NGS gene panel involving Marfan-related genes, which identified a missense variant on gene *FBN1*. After the NGS test of the index case, familial segregation analysis was performed by Sanger sequencing.

## 3 Timeline

See Figure 3.

**Figure 3:**
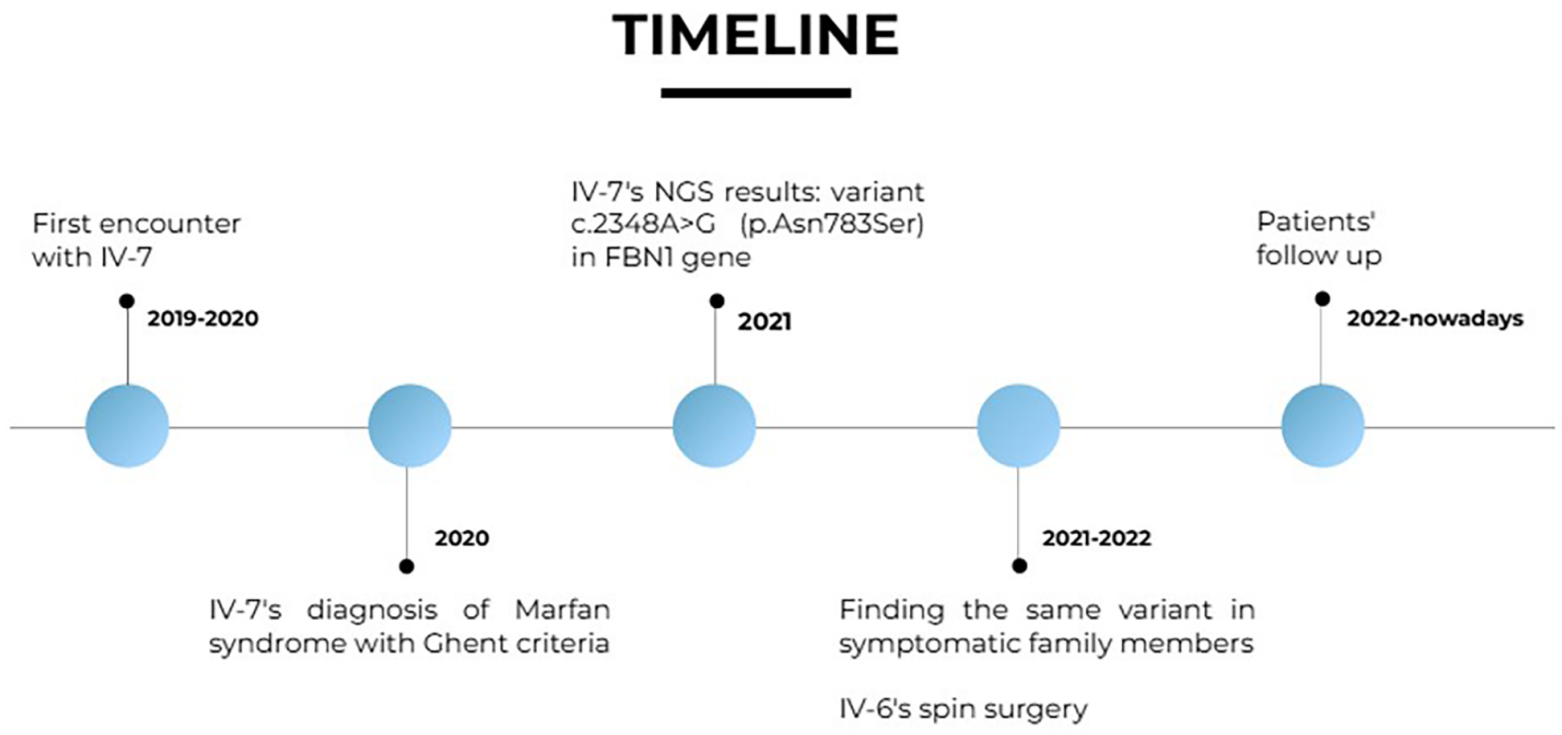
Timeline.

## 4 Diagnostic assessment

Patients were evaluated in the Pediatric Clinic and Rare Diseases, “Microcitemico Hospital”, Cagliari (Italy). All patients' data were collected from July 2019 to April 2024.

Genetic testing was performed in Medical Genetics Unit, University Hospital of Parma, Parma, Italy. Genomic DNA was extracted from peripheral blood using a QIAamp DNA Mini Kit (Qiagen, Hilden, Germany).

Next generation Sequencing of a gene panel of 5 genes (*FBN1*, *TGFBR1*, *TGFBR2*, *TGFB2*, *TGFB3*) was carried out on the proband and her parents. The coding exons were captured using the Illumina AmpliSeq focus panel and sequenced using a MiSeq sequencer (Illumina, San Diego, CA, USA) to an average depth of 100 reads per target base. Vcf files were analysed with Base Space Variant Interpreter Illumina software, aligned to a human reference genome (HG19). For variant annotation and prediction, non-synonymous substitutions and SNPs with minor allele frequencies (MAFs) lower than 5% were filtered out. Variant classification followed the ACMG/AMP 2015 guidelines (14).

The function of gene variants and their potential pathogenicity were analyzed referencing gnomAD, dbSNP, OMIM, ClinVar, LOVD, Franklin, REVEL and alphamissense tools.

Candidate variants and regions with low coverage were verified using Sanger sequencing on an Applied Biosystems 3500Dx sequencer (Applied Biosystems, Waltham, MA, USA).

According with ACMG/AMP rules, the assigned class of pathogenicity was Likely Pathogenic, based on the following scores:

• PM1: moderate (Non-truncating non-synonymous variant is located in a mutational hot spot and/or critical and well-established functional domain)
• PP2: supporting (Missense variant in a gene with low rate of benign missense mutations and for which missense mutation is a common mechanism of a disease)
• PM2: moderate (Extremely low frequency in gnomAD population databases; gnomAD there are not population frequencies)
• PP3: supporting (Extremely low frequency in gnomAD population databases)
• PP1: supporting (Cosegregation with disease in multiple affected family members in a gene definitively known to cause the disease)

## 5 Results and outcomes

**Figure 4:**
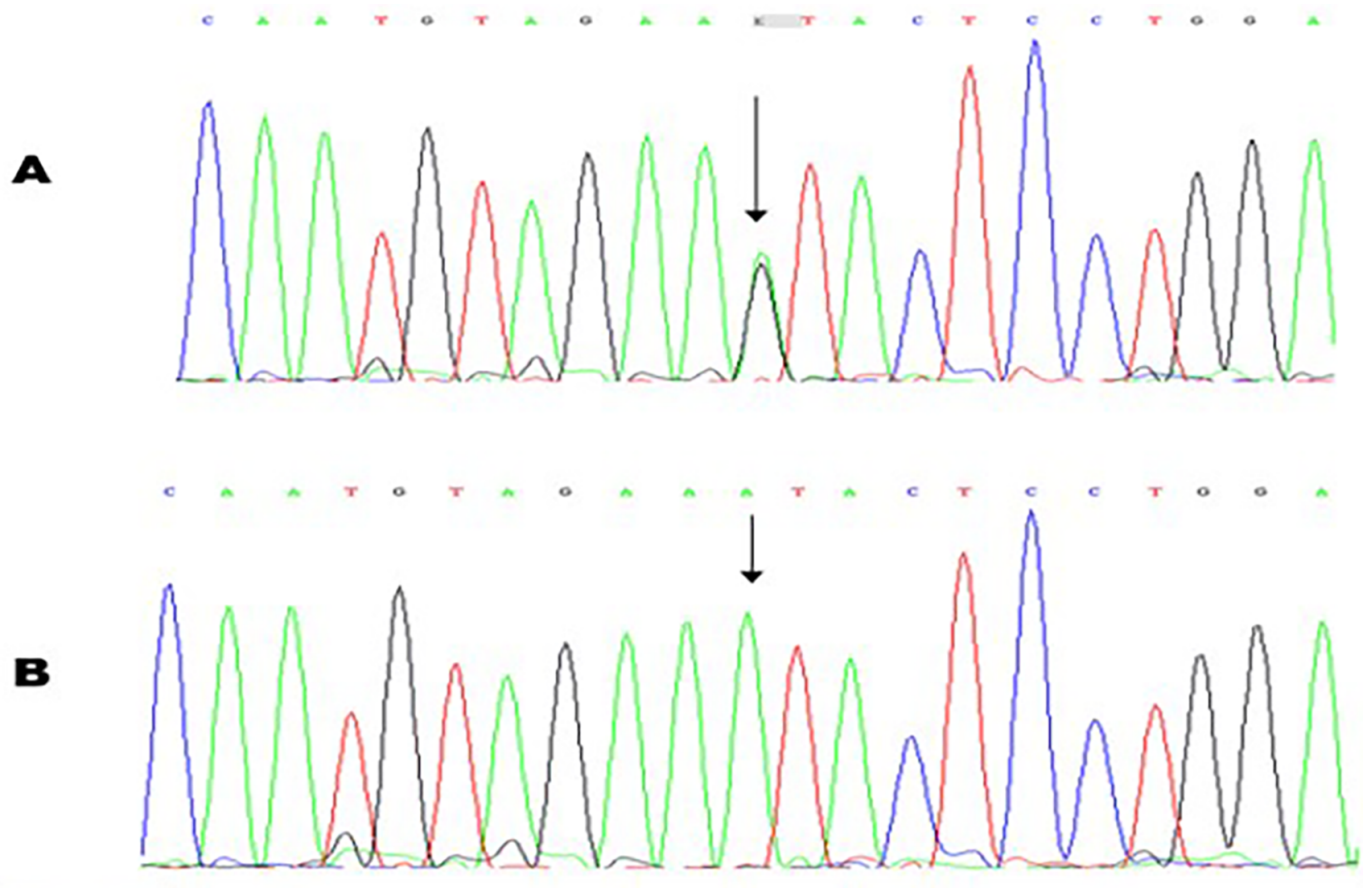
Molecular characterization of a patient with Marfan syndrome. Sequence chromatogram showing the position of the c.234A>G transversion that led to the p.Asn783Ser missense variant. The variant was heterozygous in the proband **(A)**; Sanger sequencing result of an unaffected control **(B)**.

A novel heterozygous nucleotide variant c.2348A>G (p.Asn783Ser) in exon 20 of the *FBN1* gene (see Figure 4) was at first detected in the proband and her sister (IV-6, IV-7) and subsequently in the other symptomatic family members (III-3, III-5, IV-4). No other VUS, likely pathogenic and pathogenic variants were found.

The variant NM_000138.5:c.2348A>G was not described in gnomAD Exomes tool (frequency: 0%), ClinVar and LOVD databases. The c.2348A>G variant was further investigated with the online tools REVEL and alphamissense, resulting in 0.861 and 0.902 scores, respectively; these results strongly support the pathogenicity of the *FBN1* genetic variant.

III-2, III-4, IV-5, IV-8 underwent genetic testing as well and do not carry the variant: additionally, they are in good health and they are not showing any symptoms nor signs possibly related to Marfan Syndrome.

Moreover, although II-9 is very likely a carrier of the variant, it was not possible to test her. Finally, thorough family history revealed two other relatives, I-1 and II-1, with “*ectopia lentis*”, who are thought to be affected because of their clinical history: genetic counselling could not be provided due to their passing.

All of the carriers of the variant have started their follow-up at our Rare Disease Clinic. Up to now, IV-6 has undergone orthopedic scoliosis correction in 2021. IV-7 is now enlisted for the same surgery. Both sisters are medically treated with antihypertensive medication to prevent aortic dilatation. No other treatment has been required.

## 6 Discussion and conclusions

Marfan Syndrome is a connective tissue disorder. Most of the time it is inherited with an autosomal dominant mechanism, and only 25% of cases are due to sporadic mutations (7, 8, 15). *FBN1* gene is mainly involved (4, 5).

Up to now, many variants in the *FBN1* gene have been reported regarding this syndrome (7, 16–19); the most commonly found are point mutations, of which 60% are missense and often caused by substitution of cysteine residues, which has been suggested to cause abnormal spliceosome during transcription (7, 20, 21). Nonetheless, many *FBN1* mutations could be still unknown.

Nowadays, Revised Ghent Criteria of 2010 are used to diagnose Marfan Syndrome (22, 23), as shown in Table 1.

**Table 1:** Revised Ghent nosology criteria for Marfan syndrome.

Revised Ghent Nosology	
Without family history:	With positive family history:
• Aortic root dilatation Z-score ≥ 2 AND ectopia lentis, *or*	• Ectopia lentis, *or*
• Aortic root dilatation Z-score ≥ 2 AND FBN1 mutation, *or*	• Systemic score ≥ 7 points*, *or*
• Aortic root dilatation Z-score ≥ 2 AND systemic score ≥ 7 points*, *or*	• Aortic root dilatation Z-score ≥ 2 above 20 years old, or 7 points*, *or*
• Ectopia lentis AND FBN1 mutation with known aortic root dilatation.	• Aortic root dilatation Z-score ≥ 2 below 20 years old
* Systemic score: Wrist and thumb sign (wrist OR thumb sign) Pectus carinatum (pectus excavatum OR chest asymmetry) Hindfoot deformity (plain pes planus) Pneumothorax Dural ectasia Protrusio acetabula Reduced upper segment to lower segment ratio AND increased arm/height AND no severe scoliosis Scoliosis or thoracolumbar kyphosis Reduced elbow extension Facial features (3/5) (dolichocephaly, enophthalmos, downslanting palpebral fissures, malar hypoplasia, retrognathia) Skin striae Myopia > 3 diopters Mitral valve prolapse (all types)	Points: 3 (1) 2 (1) 2 (1) 2 2 2 1 1 1 1 1 1 1

According to these criteria, diagnosis can still be made without a known *FBN1* mutation (4). In our study, a clinical diagnosis was made and it was later confirmed by genetic analysis.

As *FBN1* gene encodes for one of the main components of elastic matrix microfibrils, connective tissue is mainly involved. However, the phenotypic spectrum is very wide: given the interfamilial and intrafamilial variability, other factors, such as environmental and stochastic effects, are probably involved (7, 8, 21). Indeed, in our case variable phenotypes with different systemic complications were found.

The main symptomatology was found in both our patients and they showed two of the main typical Marfan Syndrome features: scoliosis and aortic root enlargement.

Scoliosis is usually presented at a young age and has a rapid progression (24), just like in our patients' scenario. Braces and back surgery are usually provided (25). Scoliosis could be accompanied by other musculoskeletal involvements like tall stature, pectum excavatum or carinatum, dolichostenomelia, arachnodactyly, reduced joint mobility at the elbows and digits, and joint laxity (26). Suggestive signs for MS, which were positive in our patients, are Walker-Murdoch (“wrist sign”) and Steinberg (“thumb sign”) (24, 26).

Another important feature in MS is aortic root manifestations (2, 27). They are responsible for the majority of deaths occurring in patients affected by MS (28). In order to prevent aortic dilatation, antihypertensive medications, mainly β-blockers, are used (29, 30); in addition to this, in specific cases, prophylactic aortic root replacement is performed. Other cardiovascular features include aortic dilatation and dissection, coarctation of the aorta, mitral valve prolapse (27, 30, 31). In our scenario, both IV-6 and IV-7 are treated with anti-hypertensive medication to prevent further aortic root dilatation. No cardiac surgery is planned in the near future.

Although “*ectopia lentis*” (32) usually presents in cysteine missense mutations (21), as noted by Schrijver et al. (33), in our case, it was reported in two dead relatives who, however, could not be tested for gene mutation. In our family, those patients carrying the variant do not present any typical ocular manifestations but, as recommended, periodic ophthalmic examinations are being carried out.

As previously reported, MS is a multisystemic disorder involving organs such as the nervous system (dural ectasia being present in up to 66% of cases), the muscular-skeletal system with various dysmorphisms, and other clinical manifestations (protrusion acetaboli, spontaneous pneumothorax predisposition (34) and sleep disordered breathing (35–37), glaucoma (32), which require frequent follow-up (4, 38).

Although there is not yet a clear understanding regarding the pathogenesis, two main mechanisms have been found using mouse models of MS: the first one being haploinsufficiency, a quantitative defect that leads to a deficiency of fibrillin-1, and the second one being dominant negative mutations, a qualitative defect that generates an aberrant protein with impaired function (6, 7, 39). Both mechanisms seem to play a different, yet crucial, role in the clinical development of the patients carrying the mutations.

Many studies have been brought on regarding the possibility of a correlation between genotype and phenotype, but they showed little support to this hypothesis (8). Nevertheless, some considerations may be made: for example, according to literature, there is a significantly higher risk of severe cardiac involvement, shorter survival and onset of “*ectopia lentis*” in patients with haploinsufficiency mutations, as opposed to those who carry dominant negative mutations (4). In addition to this, haploinsufficiency tends to lead to a more variable phenotype, according to the percentage of the total proteins involved in the mutation (23, 40–43). As an example, mutations in the exons 24–32 of the gene are associated with the most severe forms of Marfan syndrome, more specifically with early onset of aortic risk (22, 44). In regards to the cardiovascular features, aortic involvement seems to be more frequent when truncating and splicing mutations are found, as well as alterations in cysteine residues (45); moreover, aortic-root dilatation progressed slowly when the mutation occurred in exon 12 or 20. Truncating mutations are also associated with a lower risk of ocular involvement (41), whereas the skeletal findings and joint laxity seem to be more severe (46, 47). Finally, cardiovascular manifestations are mild, at times even absent, when exon 13 or 49 are involved (33, 44). In addition to this, non-sense mutations were never reported in cases of neonatal Marfan syndrome (22), while scoliosis occurred with higher frequency and severity, often requiring surgical intervention, when the C1-C3 or C2-C4 disulfide bonds were disrupted (7).

Early diagnosis and state-of-the-art therapies have increased life expectancy for Marfan patients up to 75 years (48, 49). Our tested patients were relatively young and as a result we managed to intervene to reduce complications or to prevent them at all, greatly improving their quality of life; for example, spine correction surgery in IV-6 has hugely contributed to her well-being, allowing her to stand in an upright position for prolonged periods of time. Moreover, our patients, will undergo periodic follow-up, which is essential to prevent complications (50): ophthalmic examination for glaucoma and cataract, orthopedic checkups in case of scoliosis, annual echocardiography or MRI to monitor aortic root enlargement and mitral prolapse.

Since *FBN1* gene mutations have mainly autosomal dominant inheritance with high penetrance, study of the probands families and their descendants is extremely important, even when they do not show any features, as to start a thorough screening process of the most common complications as soon as possible.

One limitation of our study is certainly the small number of people examined. Moreover, it was not possible to study all the members of the family. Nonetheless, it is important to highlight the absence of any pathogenic variants in the *FBN1* gene in the asymptomatic members of the family, whilst clinical manifestations were present in those who carried said variant, albeit with different levels of severity.

Surely, if the new variant is to be introduced among the already known Marfan syndrome's variants, it will be possible to substantiate our discovery and to further assess the variant's role in its phenotypic presentation.

The existence, in our structure, of the Regional Coordination Centre for Rare Diseases was fundamental. It has made it possible to rapidly extend the genetic exams to adult family members and to follow over time symptomatic patients relatively to age and clinical presentation.

In conclusion, in the present study, we identified a novel heterozygous variant of *FBN1* gene, in exon 20: a missense substitution of a serine residue with an arginine residue in the position 783 of Fibrillin-1 protein. Exon 20 is part of *FBN1*'s 5′ region and it's not frequently involved in MS. It has a complete penetrance in our family, as all of the members with this specific genotype showed a pathological—yet different—phenotype. At the same time, all the members without the variant did not present with any Marfan-related symptoms nor signs. This variant had not been reported before neither in literature nor in public databases so we believe that it should be included in genetic diagnoses and counselling discussion of families with MS.

## Data Availability

The original contributions presented in this study are included in the article. Further inquiries can be directed to the corresponding author.
